# Proteomic analysis reveals key genes and pathways involved in ovarian ageing of Qira Black sheep

**DOI:** 10.3389/fvets.2026.1860495

**Published:** 2026-06-30

**Authors:** Peilin Guo, Linlin Pei, Ningjie Liu, Wenhao Wang, Andi Qiao, Xin Xu, Chunjie Liu

**Affiliations:** 1 College of Animal Science and Technology, Tarim University, Alar, Xinjiang, China; 2 Key Laboratory of Tarim Animal Husbandry Science and Technology, Xinjiang Production & Construction Corps, Alar, Xinjiang, China; 3 College of Life Science and Technology, Tarim University, Alar, Xinjiang, China; 4 State Key Laboratory Incubation Base for Conservation and Utilization of Bio-Resources in Tarim Basin, Alar, Xinjiang, China

**Keywords:** differentially expressed proteins, ovarian ageing, proteomics, Qira Black sheep, signalling pathway

## Abstract

Ovarian senescence is the fundamental cause of reduced fertility in female animals,however, the molecular characteristics of ovarian ageing in sheep remain insufficiently defined. In this study, ovarian tissues were collected from 12 Qira Black sheep and assigned to group D (1–2 years, *n* = 6) and group H (5–6 years, *n* = 6) for LC–MS/MS-based proteomic profiling. A total of 458 differentially expressed proteins (DEPs) were identified between the two groups, including 211 upregulated and 247 downregulated proteins. Functional enrichment analyses indicated that these DEPs were mainly involved in cell-cycle regulation, oocyte maturation, amino acid metabolism, and inflammation-related signalling pathways, with the Rat Sarcoma(Ras) and Mitogen-Activated Protein Kinase Pathway(MAPK) signalling pathways showing particularly strong enrichment. Protein–protein interaction (PPI) network analysis revealed close interactions among Intraflagellar transport 80(IFT80), Insulin receptor(INSR), Angiopoietin-like 4(ANGPTL4), Receptor interacting serine/threonine kinase 3(RIPK3), Nuclear receptor corepressor 1(NCOR1), and Fms-Related Tyrosine Kinase 4(FLT4), with Insulin receptor(INSR) occupying a central hub position. Collectively, this study establishes a differential proteomic atlas of ovarian ageing in Qira Black sheep, highlights the potential importance of Rat Sarcoma(Ras) and Mitogen-Activated Protein Kinase Pathway(MAPK) signalling in this process, and identifies Insulin receptor(INSR) as a candidate target, thereby providing a theoretical basis for subsequent mechanistic studies and the development of molecular markers.

## 1 Introduction

Ovarian ageing is a key challenge in sheep reproduction and breeding. It is characterised by declines in both follicle number and quality, accompanied by endocrine dysregulation, ultimately leading to a marked reduction in the reproductive lifespan of ewes (1). Qira Black sheep, a local breed in Xinjiang, exhibit relatively strong reproductive performance and considerable genetic diversity (2). Previous studies have identified several candidate genes associated with hormone synthesis and follicular development in Qira Black sheep ovaries; however, research specifically addressing ovarian ageing in this breed remains limited. In production settings, as ewes age, oestrous expression, follicular developmental capacity, and lambing performance often deteriorate markedly, making ovarian ageing an important determinant of reduced fertility. Therefore, elucidating the molecular alterations underlying ovarian ageing in Qira Black sheep and identifying key target genes for subsequent functional validation or molecular breeding has important scientific and practical value.

The mechanisms of ovarian ageing are complex and involve the regulation of multiple pathways. Oxidative stress can promote ovarian cellular dysfunction by inducing mitochondrial damage and activating inflammatory and apoptotic pathways (3). In addition, inflammation-related signalling pathways such as TNF/NF-κB can influence ovarian cell survival and apoptosis (4). LC–MS/MS has been widely applied in proteomics owing to its deep proteome coverage and high reproducibility, providing robust technical support for investigating the potential mechanisms underlying ovarian ageing (5). Proteomics studies have been used to identify differentially expressed proteins and perform enrichment analyses across sheep with distinct fertility phenotypes (6). Moreover, proteomic profiling of ovarian tissues from single-lamb and multi-lamb ewes has enabled the identification of candidate molecular targets (7, 8).

Nevertheless, systematic investigations of ovarian ageing in sheep remain relatively scarce. The application of LC–MS/MS combined with pathway enrichment analysis is expected to facilitate the identification of key regulatory pathways involved in ovarian ageing. In the present study, Qira Black sheep were used as the experimental model, and DEPs were identified following rigorous data quality control. We aimed to characterise dynamic proteomic alterations during ovarian ageing and to provide candidate targets for understanding reproductive ageing mechanisms in sheep and for developing molecular markers for breeding, with potential long-term implications for ewe reproductive health and flock sustainability.

## 2 Materials and methods

### 2.1 Ethical approval

All animal experimental protocols were carried out in strict accordance with the relevant guidelines of the Scientific Ethics Committee of Tarim University (Approval No. PA20250312055).

### 2.2 Experimental animals and sample preparation

Ovarian tissues were collected from 12 Qira Black sheep housed at the Animal Experiment Station of Tarim University. All animals were maintained under standardized husbandry conditions and humanely sacrificed during the anestrous phase (March). Animals were anesthetized via intramuscular injection of xylazine (0.4 mg/kg), followed by euthanasia with an intravenous injection of pentobarbital sodium at a dose three times the anesthetic dosage. Death was confirmed by bilateral thoracotomy (9). The sheep were divided into two groups: a young group (Group D, n = 6, aged 1–2 years) and an aged group (Group H, n = 6, aged 5–6 years). No direct parent-offspring or full-sibling relationships existed between animals in the two groups to eliminate genetic background bias. The collected ovaries were rinsed with normal saline to remove surface blood, blotted dry with filter paper, immediately transferred to 2 mL cryotubes, snap-frozen in liquid nitrogen, and transported back to the laboratory for subsequent experiments.

### 2.3 Protein extraction

All 12 samples were ground thoroughly in liquid nitrogen. The lysis buffer consisted of 50 mM Tris (pH 7.4), 150 mM NaCl, and 1% NP-40, which was freshly prepared and supplemented with protease inhibitors before use. Lysis buffer was added at a ratio of 1 mL per 0.1 g tissue, and the homogenates were incubated on ice for 30 min. Subsequently, the lysates were centrifuged at 12,000 rpm for 10 min at 4 °C, and the supernatants were collected as total protein extracts. Protein concentrations were determined using a BCA Protein Assay Kit according to the manufacturer’s instructions (Servicebio, China). Standards and samples were dispensed into a 96-well plate, followed by 200 μL BCA working reagent per well. The plate was shaken for 30 s, incubated at 37 °C for 30 min, and absorbance was measured at 562 nm using a microplate reader. Protein concentrations were calculated from the standard curve. Protein integrity was assessed by 12% SDS–PAGE. Briefly, 100 μg protein was loaded per lane and electrophoresis was performed at 80 V for 20 min followed by 120 V for 90 min. Gels were stained with Coomassie Brilliant Blue and destained until clear bands were visible. All protein samples were stored at −80 °C until use.

### 2.4 Trypsin digestion and peptide desalting

Equal amounts of protein from each group were taken and diluted with lysis buffer to achieve identical concentrations and volumes. Dithiothreitol (DTT) was added to a final concentration of 5 mM and the mixture was incubated at 55 °C for 30 min. After cooling to room temperature, iodoacetamide (IAA) was added to a final concentration of 10 mM and samples were incubated for 15 min at room temperature in the dark. Protein precipitation was performed by adding six volumes of pre-chilled acetone and incubating at −20 °C for 4 h. Samples were centrifuged at 8,000 rpm for 10 min at 4 °C. The pellets were collected and air-dried for approximately 3 min to allow residual solvent to evaporate. Pellets were reconstituted in 50 mM NH₄HCO₃, and proteins were digested with trypsin at an enzyme-to-protein ratio of 1:50 (w/w) at 37 °C overnight. Peptides were desalted using a SOLA SPE 96-well plate (Ouyi, Shanghai, China). The plate was conditioned with 200 μL methanol twice and this conditioning procedure was repeated for three cycles. The plate was then equilibrated with 200 μL equilibration solution (0.1% formic acid in water) twice, also repeated for three cycles. After adjusting the vacuum, 100 μL of each sample was loaded at a droplet flow rate of approximately 1 mL/min (one loading step). The wells were washed with 200 μL equilibration solution twice (three cycles), and peptides were eluted with 150 μL elution solution (50% acetonitrile, 49.9% water, 0.1% formic acid) twice (three cycles). The combined eluates were vacuum-dried and stored for subsequent LC–MS/MS analysis.

### 2.5 LC–MS/MS high-resolution mass spectrometry analysis

An iRT internal standard was added to each sample. Peptides were separated on a C18 analytical column with high sensitivity and eluted using a gradient of solvent A (0.1% formic acid) and solvent B (acetonitrile) with the following programme: 6, 28, 80, and 80% B. Data-independent acquisition (DIA) was performed on a timsTOF HT mass spectrometer (Bruker, Germany) under the following settings: capillary voltage, 1.6 kV; dry temperature, 180 °C; dry gas, 3.2 L/min; mass range, 300–1,500 m/z; ion mobility, 0.7–1.3 1/K0; collision energy, 20–59 eV; and ramp time, 50 ms.

### 2.6 Protein identification and bioinformatics analysis

Raw MS data were processed using DIA-NN. Protein identification was performed against the following database: uniprot-*Ovis aries*-9940_UP000002356-irt_2024_02_02.fasta. For differential analysis, group D was set as the control group to compare protein expression profiles between the two groups. The raw data were filtered using the following criteria: Sequest HT score > 0, unique peptides ≥ 1, and either localisation probability ≥ 0.75 or Delta score ≥ 8. Missing values were filtered and imputed to obtain confident protein sites, followed by quality-control normalisation and log2 transformation. Bioinformatic analysis was performed using the OECloud tools at https://cloud.oebiotech.com. A *t*-test was used to calculate *p*-values,and statistical analysis was completed with SPSS 26.0. Proteins with *p* < 0.05 and fold change (FC) > 1.2 or FC < 0.833 were defined as differentially expressed proteins (DEPs). Gene Ontology (GO) and Kyoto Encyclopedia of Genes and Genomes (KEGG) enrichment analyses were performed for functional annotation and pathway interpretation of the DEPs. Volcano plots and hierarchical clustering heat maps were generated via OECloud online tools. Venn diagrams were generated using the Draw Venn Diagram tool. Protein–protein interaction (PPI) networks were constructed using the STRING database with the minimum interaction score set to “medium confidence” (confidence threshold > 0.4) (10).

### 2.7 Western blot

Proteins were separated by SDS–PAGE using 5% stacking gels and 12% resolving gels at 100 V for 90–120 min. PVDF membranes were pre-wetted in methanol for 2 min, and protein transfer was performed at 300–400 mA for 40–60 min. Membranes were then blocked with 5% non-fat milk at room temperature for 2 h with gentle shaking. After washing three times with TBST (5 min each), the membranes were incubated with primary antibodies overnight at 4 °C. All primary antibodies including INF2, NFKB2, BECN1, PHKA1 and PACSIN3 were purchased from Proteintech Group (Wuhan). Their catalog numbers and working dilutions were as follows: INF2 (20466-1-AP, 1:2500), NFKB2 (84022-6-RR, 1:30000), BECN1 (11306-1-AP, 1:5500), PHKA1 (24279-1-AP, 1:750), and PACSIN3 (10639-1-AP, 1:750). On the next day, the membranes were washed three times with TBST (5 min each) and incubated with secondary antibody at room temperature for 1 h. The secondary antibody was also obtained from Proteintech Group (Wuhan) with the catalog number RGAR001 at a dilution of 1:6500, followed by another three TBST washes (5 min each). Proteins were detected using enhanced chemiluminescence (ECL) in the dark and imaged using a chemiluminescence imaging system (Servicebio, China).

### 2.8 Statistical analysis

Band intensities were quantified using AIWBwell™ software. Statistical analyses were performed using SPSS 26.0. Data are presented as mean ± SD (*n* = 3). Differences between groups were assessed using an independent-samples t-test. Statistical significance is indicated as follows: *p* < 0.05 (*), *p* < 0.01 (**), and *p* < 0.001 (***).

## 3 Results

### 3.1 Quality control and protein identification

**Figure 1:**
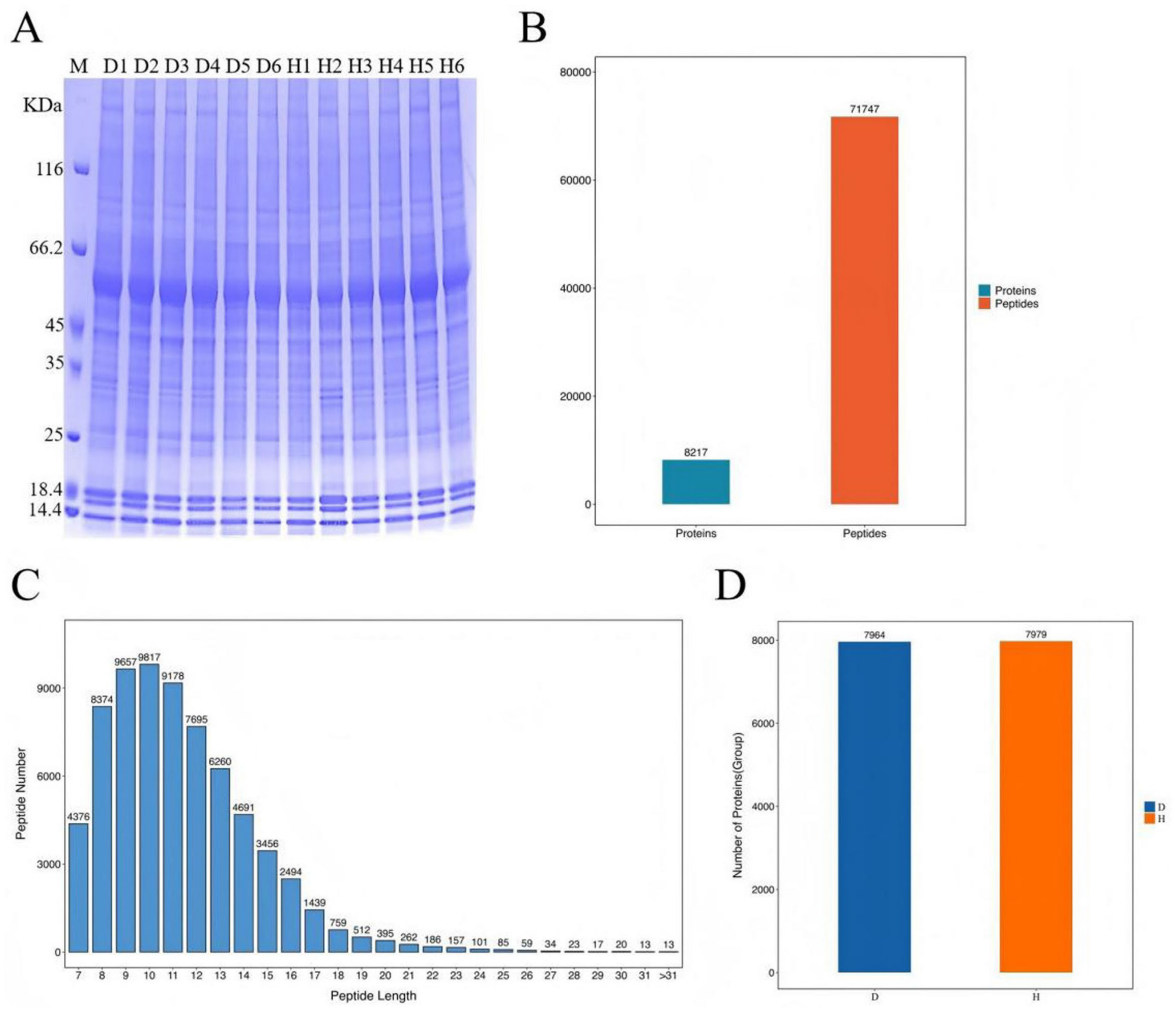
Quality control and overview of proteomic data from Qira Black sheep ovarian tissue. **(A)** SDS-PAGE analysis of extracted proteins from ovarian tissue samples. M: protein marker; lanes D1–D6: young group (1–2 years old); lanes H1–H6: old group (5–6 years old). Clear bands confirm the integrity and quality of extracted proteins for subsequent proteomic analysis. **(B)** Summary of identified peptides and proteins by LC–MS/MS. A total of 71,747 peptides and 8,217 proteins were identified across all samples. **(C)** Distribution of peptide lengths. Most identified peptides are 7–20 amino acids in length, consistent with typical tryptic peptides. **(D)** Number of reliably quantified proteins in groups D and H. Under the criterion of valid quantitative values in at least 50% of samples, 7,964 proteins (group D) and 7,979 proteins (group H) were retained for quantitative analysis.

As shown in Figure 1A, clear protein bands were observed in all groups, indicating that the extracted proteins were of sufficient quality for proteomic analysis. LC–MS/MS identified a total of 71,747 peptides and 8,217 proteins (Figure 1B). Most peptides were 7–20 amino acids in length (Figure 1C). Proteins with valid quantitative values in at least 50% of samples were considered reliably quantified; under this criterion, 7,964 and 7,979 proteins were retained for quantification in groups D and H, respectively (Figure 1D).

### 3.2 Identification of DEPs

**Figure 2:**
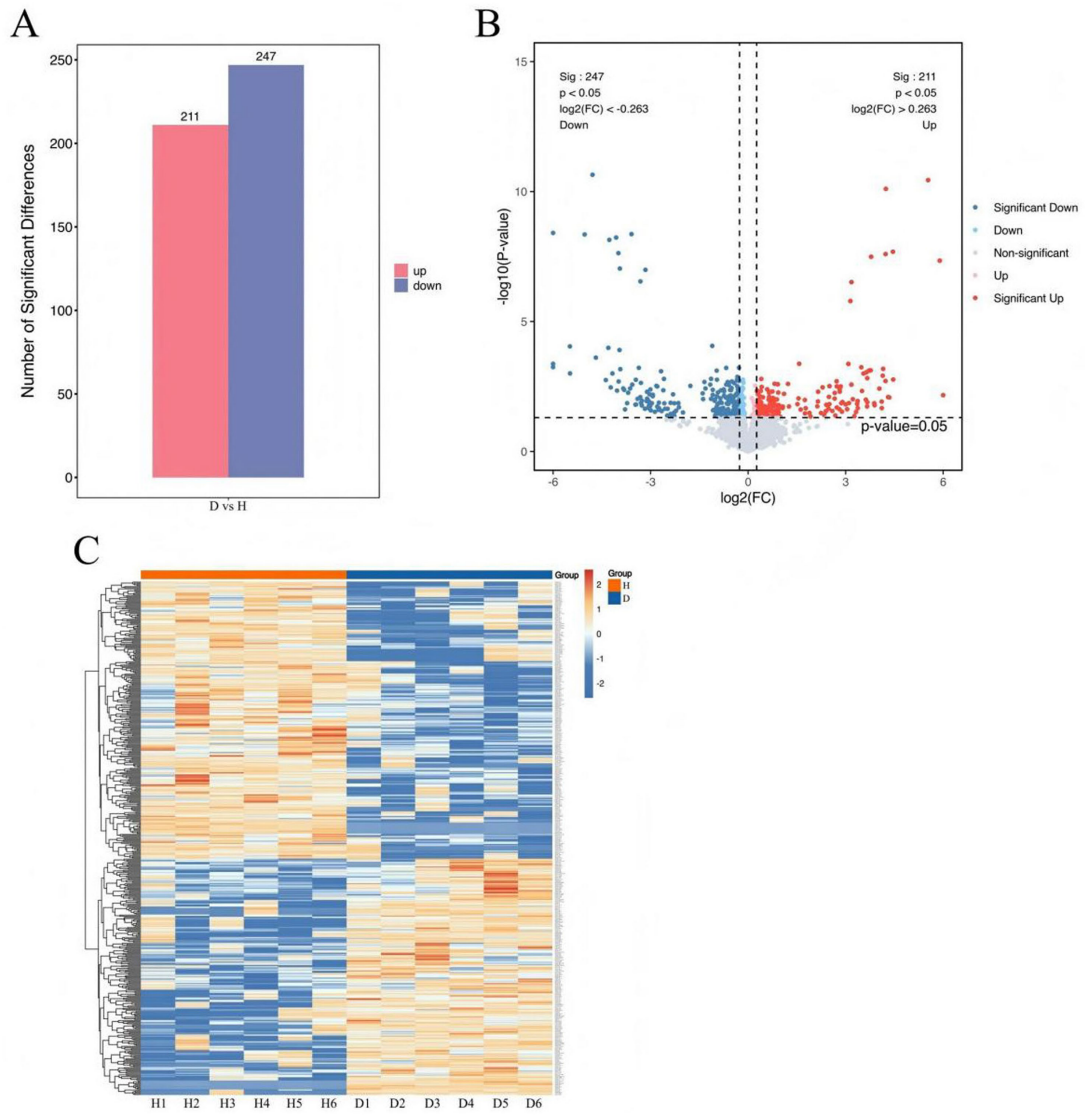
Identification and hierarchical clustering of differentially expressed proteins (DEPs) between groups D and H. **(A)** Number of upregulated and downregulated DEPs. A total of 458 DEPs were identified, including 211 upregulated and 247 downregulated proteins. **(B)** Volcano plot of DEPs. Significantly upregulated (red, log₂(FC) > 0.263, *p* < 0.05) and downregulated (blue, log₂(FC) < −0.263, *p* < 0.05) proteins are highlighted. **(C)** Hierarchical clustering heatmap of DEPs. Samples are clustered according to their group identity (D: young group, H: old group), demonstrating high within-group similarity and distinct between-group separation.

A total of 458 differentially expressed proteins (DEPs) were screened between group D (control) and group H. Among them, 211 proteins were significantly upregulated and 247 were downregulated in group H compared with group D (Figures 2A,B). Hierarchical clustering analysis of all DEPs showed high similarity of protein expression profiles within each group and obvious separation between the two groups, which was consistent with the experimental expectation (Figure 2C).

### 3.3 GO enrichment analysis of DEPs

**Figure 3:**
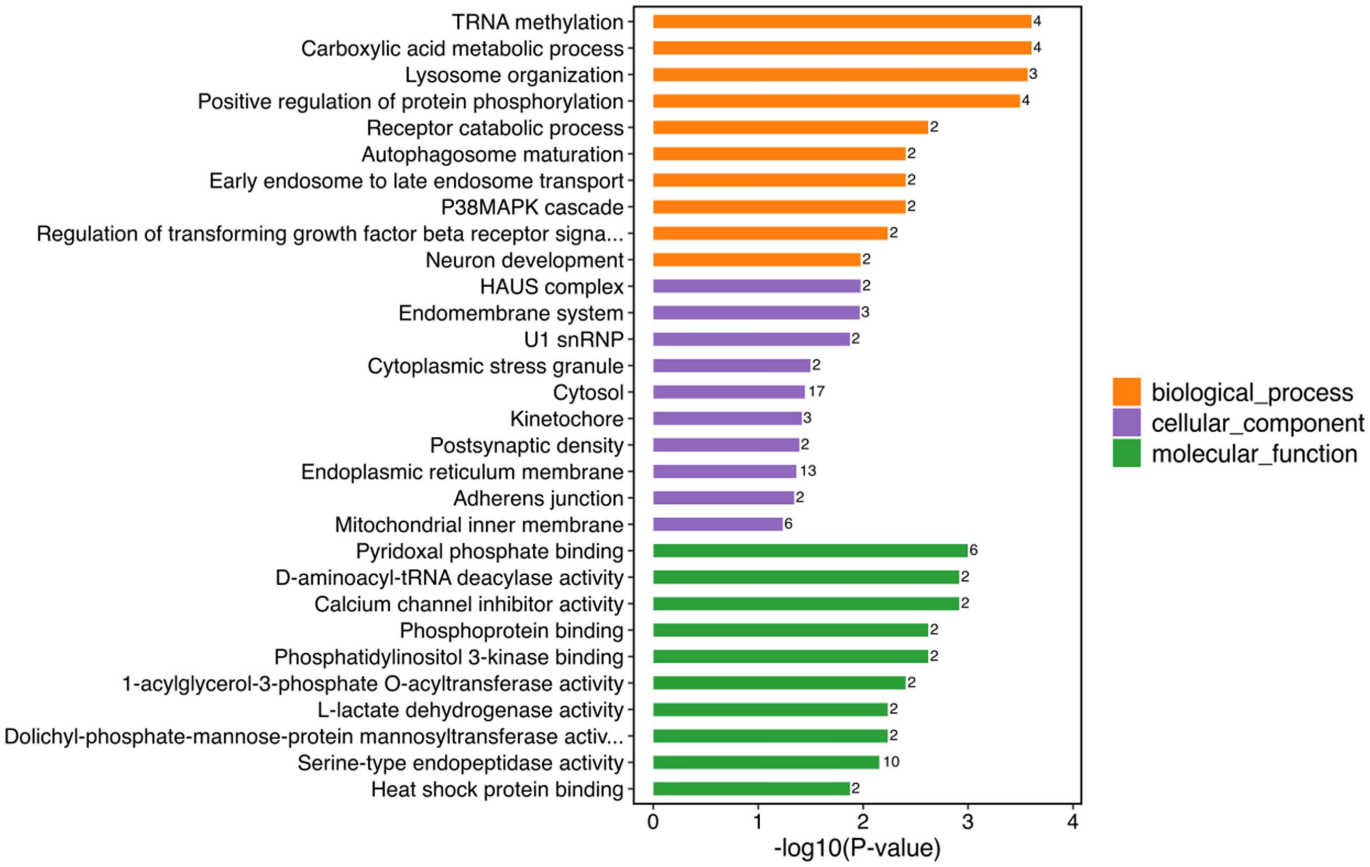
GO enrichment analyses of differentially expressed proteins (DEPs) between groups D and H. DEPs are categorized into biological process (BP), cellular component (CC), and molecular function (MF). Key enriched terms include tRNA methylation, positive regulation of protein phosphorylation, p38 MAPK cascade, and regulation of transforming growth factor beta receptor signalling pathway.

GO enrichment analysis indicated that DEPs were significantly enriched in the Biological Process (BP) category, including tRNA methylation, positive regulation of protein phosphorylation, p38 MAPK cascade, and regulation of transforming growth factor beta receptor signalling pathway (Figure 3). In the Cellular Component (CC) category, significant enrichment was observed for the HAUS complex, endomembrane system, and cytoplasmic stress granule. In the Molecular Function (MF) category, enriched terms included phosphatidylinositol 3-kinase binding, L-lactate dehydrogenase activity, and serine-type endopeptidase activity.

### 3.4 KEGG pathway enrichment analysis of DEPs

**Figure 4:**
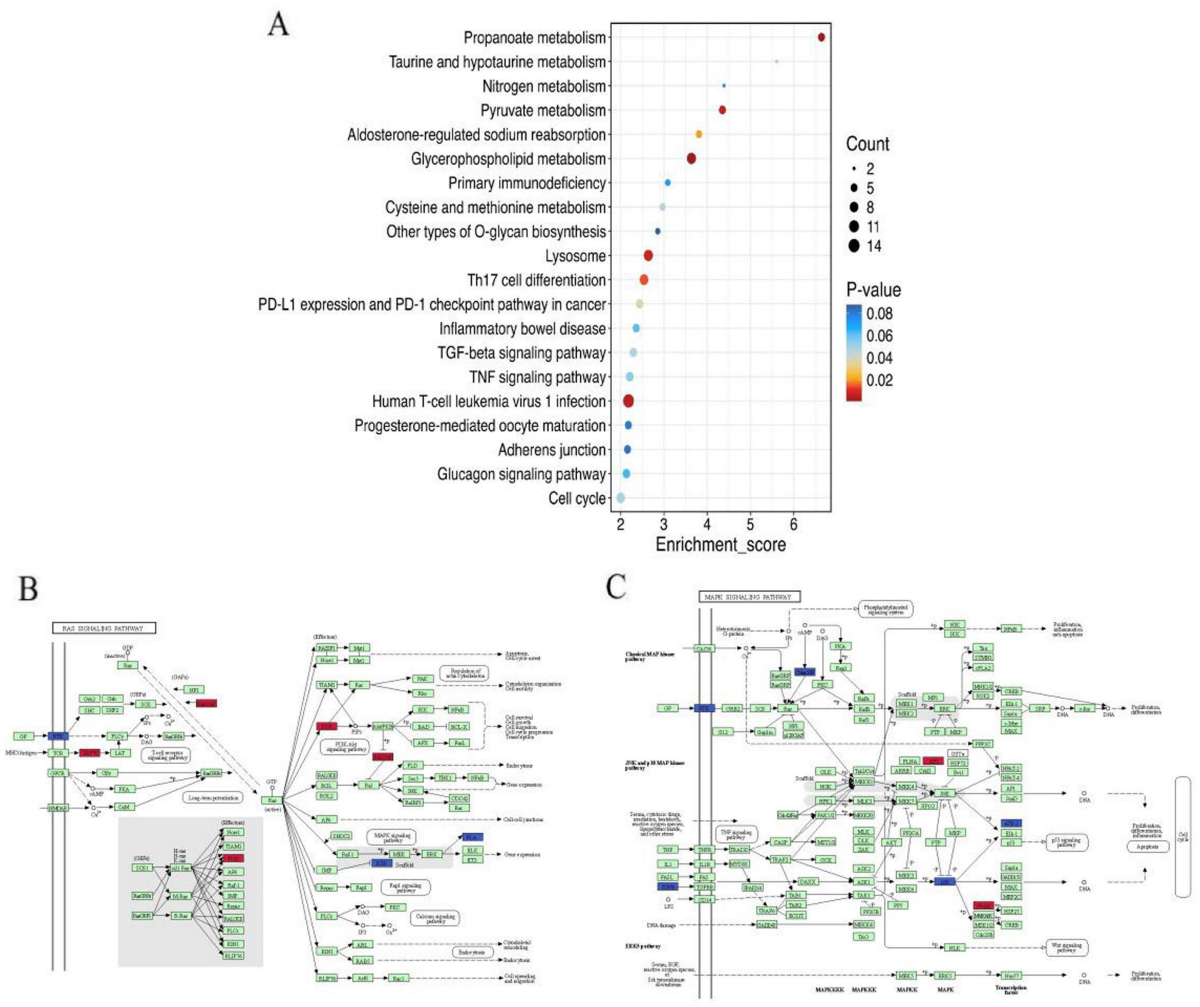
KEGG pathway enrichment analyses of differentially expressed proteins (DEPs) between groups D and H. **(A)** KEGG pathway enrichment scatter plot of DEPs. The top enriched pathways include Cell cycle, Glucagon signalling pathway, Progesterone-mediated oocyte maturation, TNF signalling pathway, and Cysteine and methionine metabolism. **(B)** Visualization of the Ras signalling pathway. Upregulated DEPs are highlighted in red, and downregulated DEPs are highlighted in blue. **(C)** Visualization of the MAPK signalling pathway. Upregulated DEPs are highlighted in red, and downregulated DEPs are highlighted in blue.

KEGG enrichment analysis annotated 279 pathways. In this study, the top 20 pathways with the smallest *p*-values were selected to generate the bubble plot. As shown in Figure 4A, DEPs were mainly involved in pathways including Cell cycle, Glucagon signalling pathway, Progesterone-mediated oocyte maturation, TNF signalling pathway, and Cysteine and methionine metabolism (Figure 4A). Further screening revealed that the Ras signalling pathway and MAPK signalling pathway were the two pathways with the largest number of annotated DEPs. In the Ras signalling pathway, LOC101112639, RALGAPA2, RASAL1, and PIK3R2 were upregulated, whereas INSR, FLT4, KSR1, and PLAAT3 were downregulated (Figure 4B). In the MAPK signalling pathway, MAPKAPK5 and MAPK8IP3 were upregulated, while TGFB1, ATF2, INSR, FLT4, RAPGEF2, and MAPK12 were downregulated (Figure 4C).

### 3.5 PPI network of DEPs

**Figure 5:**
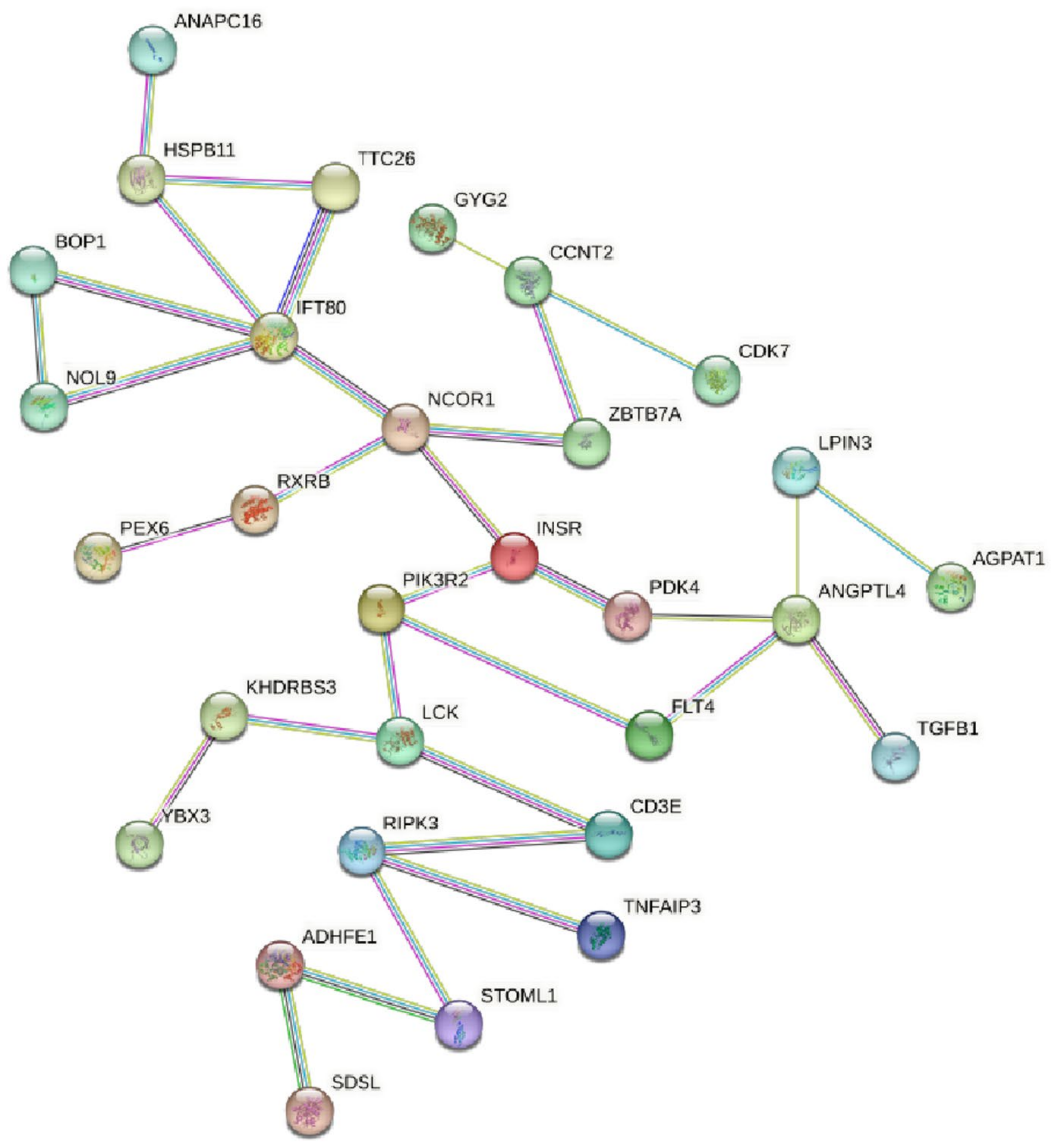
Protein–protein interaction (PPI) network of key DEPs. PPI network of DEPs. Key hub proteins include IFT80, INSR, ANGPTL4, RIPK3, NCOR1, and FLT4, with INSR acting as a central node connecting multiple signalling and metabolic pathways.

As shown in Figure 5, several DEPs—including IFT80, INSR, ANGPTL4, RIPK3, NCOR1, and FLT4—showed strong interactions within the PPI network. Distinctly, as a key upstream regulator of the Ras and MAPK signalling pathways, INSR occupied a central hub position and linked multiple signalling and metabolic regulators.

### 3.6 Western blot

**Figure 6:**
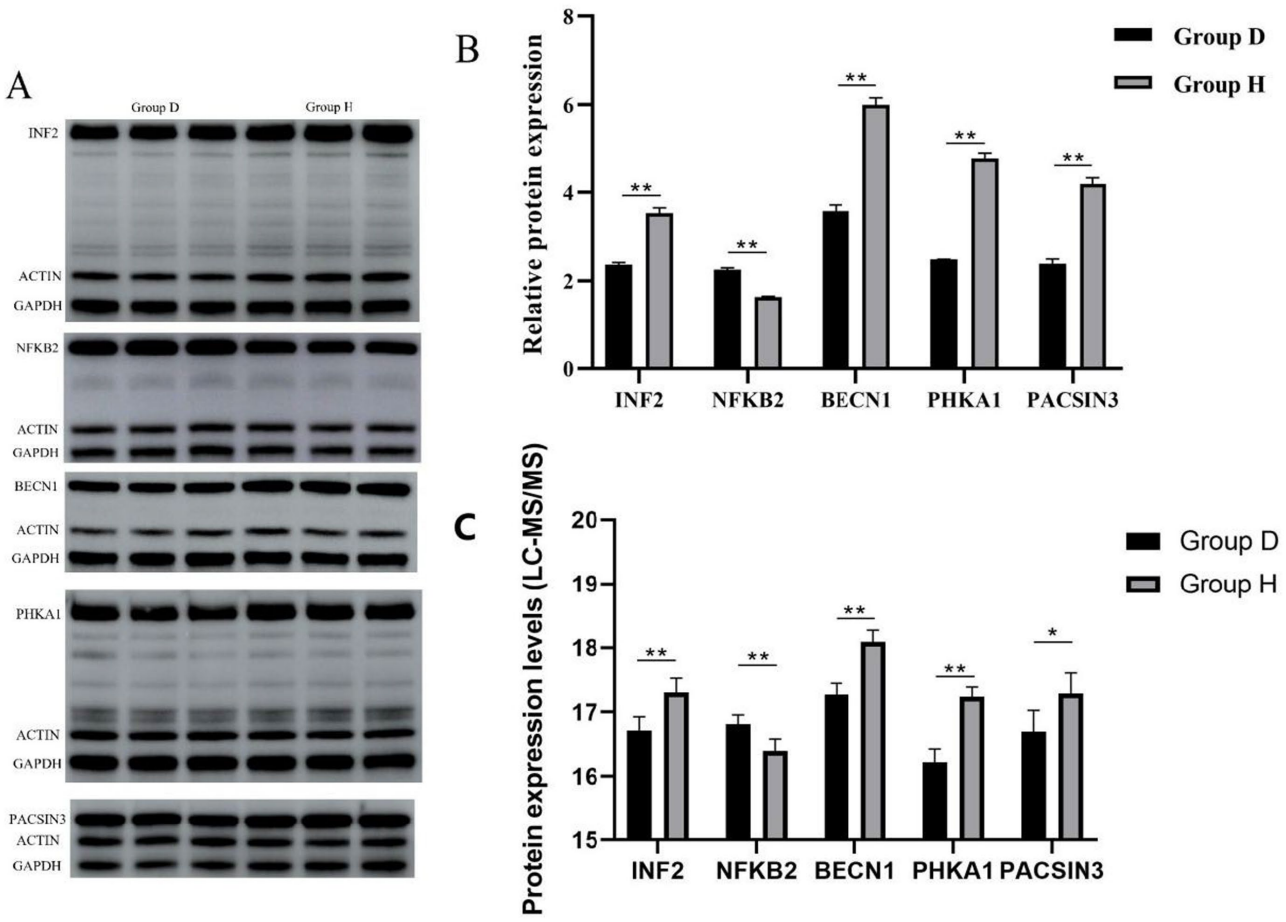
Western blot and LC–MS/MS validation of selected differentially expressed proteins (DEPs). **(A)** Representative Western blot images of INF2, NFKB2, BECN1, PHKA1, and PACSIN3 in Group D and Group H. ACTIN and GAPDH were used as loading controls. **(B)** Quantification of relative protein expression based on Western blot analysis. **(C)** LC–MS/MS quantification of protein expression levels. The expression trends of all five proteins were consistent between the two methods: INF2, BECN1, PHKA1, and PACSIN3 were upregulated in Group H, while NFKB2 was downregulated. Data are presented as mean ± SD, *n* = 6 per group. *p < 0.05, **p < 0.01 vs. Group D.

Five proteins were selected for validation by Western blotting. INF2, BECN1, PHKA1, and PACSIN3 were upregulated in ovaries from group H, whereas NFKB2 was downregulated. These expression trends were consistent with the LC–MS/MS results, supporting the reliability of the proteomic data (Figures 6A–C).

## 4 Discussion

Sheep ovarian ageing is closely associated with a wide range of cellular functions and biological processes (11). In this study, ovarian tissues were collected from 12 Qira Black sheep, including 6 young individuals and 6 aged individuals, which were divided into group D and group H. Total proteins were extracted and analyzed via LC–MS/MS high-resolution mass spectrometry. A total of 458 DEPs were identified in group H compared with group D, consisting of 211 upregulated proteins and 247 downregulated proteins. Accordingly, GO enrichment analysis was performed to explore pathway differences between groups D and H. DEPs were enriched in terms such as tRNA methylation, p38MAPK cascade, regulation of transforming growth factor beta receptor signalling pathway, carboxylic acid metabolic process, L-lactate dehydrogenase activity, and pyridoxal phosphate binding. These findings indicated that ovarian ageing altered cell growth cycle, energy substrate utilization and cofactor requirement, thereby affecting cellular development and metabolic function. In addition, DEPs were involved in multiple cell division-related pathways, which may contribute to impaired follicular development and reduced oocyte maturation through changes in ovarian cell proliferation and apoptosis. This is consistent with previous studies indicating that ovarian ageing is characterised by DNA damage and cell-cycle dysregulation (12, 13). Enrichment of DEPs in postsynaptic density and neuron development may reflect alterations in ovarian neural signalling network and neuronal development, with potential implications for the ovary–hypothalamus–pituitary axis (14). Neuronal metabolism has also been reported to exert specific effects on ovarian fertility (15).

KEGG enrichment further indicated that DEPs were involved in inflammatory and immune-related pathways, including the TNF signalling pathway and TGF-beta signalling pathway, implying immune dysregulation and chronic inflammatory responses during ovarian ageing. The enrichment of metabolic pathways such as Pyruvate metabolism and Taurine and hypotaurine metabolism, together with the upregulation of related proteins in group H, suggests increased antioxidant capacity during ovarian ageing. Age-associated declines in energy metabolic efficiency and increased oxidative stress have been implicated in reduced oocyte quality and ovarian functional deterioration (16). Moreover, enrichment of the Cell cycle pathway, with several cell-cycle-related proteins downregulated in group H, may indicate diminished ovarian reserve and growth capacity, collectively highlighting a potential role of oxidative stress in reproductive ageing.

Ras and MAPK are core pathways regulating stress responses, proliferation, and differentiation in the ovary (17, 18) and are involved in granulosa cell (GC) proliferation and differentiation, steroidogenesis, and ovulation (19). Under oxidative stress, MAPK signalling can mediate GC apoptosis and cellular senescence, thereby promoting follicular atresia and accelerating ovarian functional decline (20). In the present study, the Ras signalling pathway and MAPK signalling pathway possessed the largest number of annotated DEPs, and INSR was downregulated in both. INSR is a receptor tyrosine kinase that can influence cellular proliferation and differentiation via metabolic signalling pathways, including Ras/MAPK (21). Previous studies have shown that the insulin receptor is expressed in granulosa cells and follicles across multiple species, highlighting its importance in follicular growth and ovulation (22, 23). Based on current proteomic observations, it can be preliminarily inferred that INSR downregulation may weaken the transduction efficiency of insulin and metabolic signalling pathways, disrupt ovarian cellular metabolic homeostasis, and ultimately trigger ovarian ageing.

In summary, all ovarian samples were collected uniformly in March, when experimental sheep undergo natural seasonal anestrus. Standardized sampling eliminates individual variations derived from fluctuating estrous cycles, unifies the physiological background across groups, and facilitates accurate identification of age-driven proteomic alterations. This study systematically characterizes proteomic signatures associated with ovarian ageing in Qira Black sheep and identifies multiple critical pathways and candidate targets. However, certain limitations exist: the present data only describe the proteomic profile of photoperiod-regulated quiescent ovaries during anestrus, and the expression patterns of screened DEPs and relevant pathways remain unknown in the breeding season. Further sampling across distinct reproductive stages combined with cellular functional assays is required to validate prospective therapeutic targets and elaborate the molecular regulatory mechanisms underlying ovine ovarian ageing.

## 5 Conclusion

In this study, we used proteomic profiling to analyse ovarian protein expression in Qira Black sheep at a young stage (1–2 years) and an aged stage (5–6 years). A total of 458 DEPs were identified. These DEPs were predominantly enriched in processes related to cell-cycle regulation, antioxidant modulation, and inflammatory/immune signalling, with the Ras and MAPK signalling pathways representing the two most enriched pathways. Furthermore, PPI network analysis highlighted INSR as a central hub protein, suggesting it as a key candidate target for subsequent validation of ovarian ageing.

## Data Availability

The mass spectrometry proteomics data have been deposited to the iProX repository under project ID IPX0016405000 and are accessible via ProteomeXchange (https://proteomecentral.proteomexchange.org) with identifier PXD076327.
